# An oldie but a goodie: Methenamine as a nonantibiotic solution to the prevention of recurrent urinary tract infections

**DOI:** 10.1371/journal.ppat.1011405

**Published:** 2023-06-15

**Authors:** Cindy Gu, A. Lenore Ackerman

**Affiliations:** Department of Urology, Division of Pelvic Medicine and Reconstructive Surgery, David Geffen School of Medicine at UCLA, Los Angeles, California, United States of America; Tufts Univ School of Medicine, UNITED STATES

## Introduction

Urinary tract infections (UTIs) are one of the most common adult bacterial infections. The global burden of UTIs has nearly doubled in the last 30 years with more than 400 million affected individuals in 2019 [1]. High UTI prevalence accounts for a significant proportion of outpatient and emergency visits, incurring billions in United States healthcare expenditures [2–4]. Rates of UTI recurrence within 6 to 12 months of the initial episode range from 25% to 44% [5,6]. Recurrent UTIs (rUTIs), defined as 2 or more symptomatic UTIs in 6 months or 3 or more episodes in a year, impose significant clinical challenges for clinicians and patients.

Despite the growing financial and societal burden of rUTIs, there has been little innovation in prevention or treatment over the past decade [7]. Both initial and recurrent UTIs are commonly managed with intermittent antibiotic treatment of individual episodes, making UTIs the second most common indication for antibiotic prescriptions and accounting for 15% of antibiotic prescriptions overall [4,8]. While multiple guidelines suggest alternative preventive regimens for rUTIs [9], the standard prophylactic regimen still involves daily antibiotics. With increasing antibiotic use and limited innovation, however, multidrug resistance (MDR) is now a global public health threat [10]. Nonantibiotic options for UTI management are needed to combat rising antimicrobial resistance and decrease rUTI burden.

### Methenamine pharmacology

**Fig 1:**
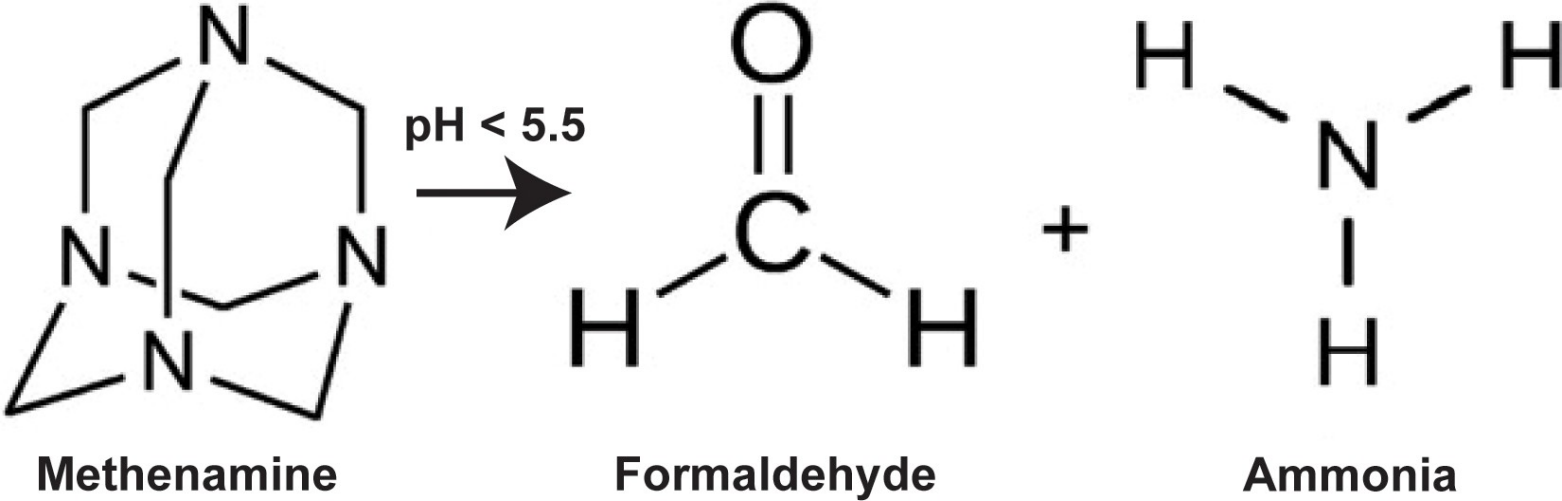
Methenamine pharmacology. Methenamine is a heterocyclic organic compound with a cage-like structure. In acidic environments, it is metabolized to formaldehyde and ammonia, which is bacteriostatic in the urinary tract.

Methenamine is a urinary antiseptic first introduced in 1895 [11]. In an acidic environment, methenamine is converted to ammonia and formaldehyde (Fig 1), which inhibits prokaryotic cell division and denatures bacterial proteins and nucleic acids [12]. Despite over a century of use, there is no evidence of bacterial resistance to methenamine’s bacteriostatic activity. Because of the known link between formaldehyde and cancer (specifically nasopharyngeal cancer or leukemia), there have been concerns about methenamine’s carcinogenic potential. Although no studies have looked directly at the long-term effects, no case reports document cancer arising as a result of methenamine use. Also, animal models have shown no evidence of carcinogenicity or increase in neoplasm rates when given methenamine orally [13].

Methenamine is available in 2 salt forms—methenamine hippurate (MH) and methenamine mandelate. Methenamine mandelate salt tabs, which have an enteric coating, are dosed 4 times daily at 1 g. MH is only available without an enteric coating and is dosed at 1 g twice daily, although higher doses can be used. As a result of these dosing schedules, MH is the more commonly used formulation. Methenamine has excellent oral bioavailability with 70% to 90% being renally secreted [14] and is safe to use during pregnancy. The primary disadvantage of methenamine is gastrointestinal (GI) side effects, such as stomach upset, cramps, and decreased appetite [15], although symptoms are typically minimal and can be countered by taking the medication with food. Few serious side effects are reported. While hemorrhagic cystitis has been documented as a rare side effect, the only case was reported 50 years ago in the context of pediatric methenamine overdose, which resolved spontaneously without long-term sequelae [16]. Patients may complain about the chemical taste or large pill size; however, MH can be cut or crushed and mixed with a flavored drink or applesauce if needed.

Due to its mechanism of action, concurrent treatment with MH and urinary acidifiers, such as ascorbic acid (Vitamin C), had been recommended to enhance methenamine efficacy. Urinary acidifiers, however, have not been found to have a significant mean effect on urine pH [17] and, in randomized controlled trials, demonstrate no evidence of enhanced effect [18,19]. As patients can complain of worsening GI side effects with these supplements, they are no longer recommended.

Despite its low side effect profile, methenamine fell out of favor in the late 20th century with the discovery and expansion in use of antibiotics. Given the growing crisis of antibiotic resistance, there has been a recent resurgence in methenamine use as a viable alternative to antibiotics for UTI prevention.

### Methenamine versus antibiotics in UTI prevention

In 2012, a Cochrane review analyzed the available trials evaluating the efficacy of methenamine for UTI prevention [20]. The primary outcome was the proportion of patients with cystitis symptoms and a positive urine culture. Although symptomatic UTI was a measured endpoint, the bacteriological criteria for UTI were not adequately described in all studies. While this study suggested that MH may be effective for UTI prevention in individuals without renal tract anomalies, the poor quality of the analyzed studies highlighted the need for higher-quality trials.

Since the Cochrane review, 2 randomized trials have evaluated the efficacy of methenamine compared to daily prophylactic antibiotics in preventing rUTIs. Both studies excluded patients with urinary tract abnormalities and/or neurogenic voiding dysfunction. The multicenter, randomized, open label, noninferiority ALTAR trial compared MH to low-dose antibiotic prophylaxis with either nitrofurantoin, trimethoprim, or cephalexin for 12 months [21]. Their primary endpoint was self-reported symptomatic UTIs requiring antibiotic treatment. The incidences of antibiotic-treated UTI episodes over the year were similar between the MH and antibiotic groups (1.4 versus 0.9 episodes/year), demonstrating noninferiority. While baseline antibiotic resistance rates were similar between the 2 groups, during treatment, higher proportions of antibiotic resistance were seen in the antibiotic arm (72%; 46/64) compared to the MH arm (56%; 39/70, *p* = 0.05). Six months after discontinuing preventive treatment, both groups exhibited similar, reduced proportions of resistant *Escherichia coli* isolates of 38% (15/39) in the antibiotic arm and 42% (19/45) in the MH arm (*p* = 0.73). Despite a lower proportion of antibiotic resistance, the MH cohort demonstrated higher rates of asymptomatic bacteriuria (14% versus 7%) at 12 months. Of participants with *E*. *coli* isolated at baseline and at least 1 postbaseline sample, similar proportions of participants (52% versus 36%) in the antibiotic and MH arms developed new resistance to at least 1 antibiotic (*p* = 0.1). The acquisition of *E*. *coli* MDR at any follow-up was also similar in both arms, at 17% (7/42) and 14% (6/42) of participants in the antibiotic and MH arms (*p* = 0.76), respectively. Six months posttreatment, however, a higher proportion of participants in the MH arm (20%; 9/45) demonstrated *E*. *coli* MDR than in the antibiotic arm (5%; 2/39, *p* = 0.06). As fewer than half of participants were assessed at this time point, it is unclear if this difference reflects higher incidence of acutely treated UTIs throughout the study, the bacteriostatic nature of MH, or sampling bias.

Botros and colleagues conducted a single-center, randomized, open-label, noninferiority trial comparing MH to trimethoprim prophylaxis for treatment duration of 6 months [22]. Their primary outcome was culture-proven UTI recurrence by 12 months. There were no differences between the MH and trimethoprim prophylaxis groups in either number of UTI recurrences or time to subsequent UTI infection (100 days versus 119 days). As in the ALTAR trial, patients in the trimethoprim arm developed increased antibiotic resistance (80%) in comparison to the MH arm (38%), despite equivalent baseline resistance rates. These 2 randomized controlled trials highlight that MH is noninferior to daily antibiotic prophylaxis in the management of rUTIs without significant adverse effects or clear increases in antibiotic resistance.

### Methenamine and host microbial communities

Although previously considered sterile, sensitive molecular methods, such as next-generation sequencing (NGS) and multiplex polymerase chain reaction (PCR), have revealed that the urinary tract contains commensal microbial communities even in healthy, asymptomatic individuals [23,24]. Disruption of these microbial communities has been implicated in rUTI risk [25]. Antibiotic use can have a profound and lasting effect on commensal microbial communities. Repeated antibiotics can alter vaginal microbiota, leading to loss of commensal *Lactobacillus* spp. and the expansion of pathogenic species such as *Gardnerella*, which increases the risk of rUTI [26]. Shifts in GI microbiota are noted only 3 days after initiating an antibiotic [27], with changes lasting up to 1 year after repeat exposures [28]. While unstudied, the urobiome would be anticipated to exhibit similar microbial shifts in response to antibiotics.

As its mechanism of action is primarily bacteriostatic, methenamine may preserve protective microbial diversity. Recently, Acevedo-Alvarez and colleagues evaluated the longitudinal effect of MH on the urobiome of 6 postmenopausal women with rUTIs [29]. From each participant, they obtained voided and catheterized urine samples as well as periurethral swabs daily for 1 week before and 3 months after starting MH. MH treatment increased the richness of the urobiome in catheterized urine specimens with little change in evenness or overall diversity. Although longer-term treatment durations not assessed, the preservation of microbial diversity with MH is encouraging.

## Conclusions

The growing crisis of antibiotic resistance has led to a reintroduction of methenamine as a viable and efficacious nonantibiotic management strategy for UTI prevention. Methenamine is as effective in UTI prevention as prophylactic antibiotics with a low side effect burden in randomized, controlled trials. Despite over a century of use, no bacterial resistance mechanisms or increased carcinogenesis have been observed. Although methenamine is gaining ground for UTI prevention, future studies will need to evaluate its longitudinal effect on host microbiota, both in and outside the urinary tract, and long-term impacts of such changes on subsequent health and disease. Given a new appreciation of the collateral damage of repeated antibiotic use on human health, effective nonantibiotic therapies, such as methenamine, must be revisited as part of the armamentarium in UTI prevention.
